# AIMTOR, a BRET biosensor for live imaging, reveals subcellular mTOR signaling and dysfunctions

**DOI:** 10.1186/s12915-020-00790-8

**Published:** 2020-07-03

**Authors:** Nathalie Bouquier, Enora Moutin, Lionel A. Tintignac, Amandine Reverbel, Elodie Jublanc, Michael Sinnreich, Yan Chastagnier, Julien Averous, Pierre Fafournoux, Chiara Verpelli, Tobias Boeckers, Gilles Carnac, Julie Perroy, Vincent Ollendorff

**Affiliations:** 1 IGF, University of Montpellier, CNRS, INSERM, Montpellier, France; 2 University Hospital Basel, Department of Biomedecine, Basel, Switzerland; 3 DMEM, University of Montpellier, INRAE, Montpellier, France; 4 Université Clermont Auvergne, INRAE, Unité de Nutrition Humaine, UMR1019, Clermont-Ferrand, France; 5 Cnr Institute of Neuroscience, Via Vanvitelli, 3220129 Milan, Italy; 6 Anatomie und Zellbiologie Universität Ulm, Albert-Einstein Allee 11, Raumnummer 4105, M24, 89081 Ulm, Germany; 7 Phymedexp, University of Montpellier, CNRS, INSERM, Montpellier, France

**Keywords:** mTor signaling, mTORC1 Biosensor, BRET, Muscle differentiation, mToropathies, Neuronal activity, Autism spectrum disorder

## Abstract

**Background:**

mTOR signaling is an essential nutrient and energetic sensing pathway. Here we describe AIMTOR, a sensitive genetically encoded BRET (Bioluminescent Resonance Energy Transfer) biosensor to study mTOR activity in living cells.

**Results:**

As a proof of principle, we show in both cell lines and primary cell cultures that AIMTOR BRET intensities are modified by mTOR activity changes induced by specific inhibitors and activators of mTORC1 including amino acids and insulin. We further engineered several versions of AIMTOR enabling subcellular-specific assessment of mTOR activities. We then used AIMTOR to decipher mTOR signaling in physio-pathological conditions. First, we show that mTORC1 activity increases during muscle cell differentiation and in response to leucine stimulation in different subcellular compartments such as the cytosol and at the surface of the lysosome, the nucleus, and near the mitochondria. Second, in hippocampal neurons, we found that the enhancement of neuronal activity increases mTOR signaling. AIMTOR further reveals mTOR-signaling dysfunctions in neurons from mouse models of autism spectrum disorder.

**Conclusions:**

Altogether, our results demonstrate that AIMTOR is a sensitive and specific tool to investigate mTOR-signaling dynamics in living cells and phenotype mTORopathies.

## Background

In eukaryotic cells, the mTOR kinase signaling serves as a nutrient, growth factor, and energetic sensing pathway crucial for cell growth and metabolism [1, 2]. mTOR-signaling activation induces anabolic processes including protein, lipid, and nucleotide syntheses while suppressing autophagy and also directs energetic metabolic cell adaptations by stimulating glycolysis and mitochondria biogenesis. In order to maintain tissue homeostasis, multiple regulation mechanisms exist to coordinate mTOR activation and macromolecule syntheses with several external cues including the availability of nutrients, the presence of growth factors, and the energetic status [3].

The mTOR kinase belongs to two main multiprotein complexes named mTORC1 and mTORC2 that differ in their protein composition, activity, and substrate specificity [3, 4]. mTORC1 is primarily involved in anabolic pathways and notably stimulates the initiation and elongation of protein translation by phosphorylating specific substrates such as 4EBP1 and S6K1 [5, 6]. Additionally, mTORC1 inhibits autophagy and lysosome biogenesis through direct phosphorylation of ULK1 and TFEB, respectively [7, 8]. Among other functions, mTORC2 controls several metabolism adaptations like cell glucose entry and glycogen synthesis via phosphorylation of its substrate Akt [9].

Several studies have shown that full mTORC1 activation occurs mainly at the surface of the lysosome and depends at least on two complementary parallel cascades of events [10]. On one side, a nutrient-dependent route involves a multiprotein signaling complex required for mTOR to sense the presence of amino acids [11]. On the other side, an essential growth factor PI3K/Akt-dependent activation pathway releases mTOR from the inhibition of the tuberous sclerosis complex by phosphorylating it [12]. This leads to the activation of the small Rheb GTP protein required for full mTORC1 activation [13]. Outside this central lysosomal platform required for mTORC1 activation, other subcellular compartments have been associated with mTOR activity including the peroxisome, the cytoplasm, and the nucleus and close to the mitochondria [14]. However, the importance of these alternative subcellular localizations for mTOR to exert its activity and their relative contribution to mTOR cell signaling integration remain to be fully determined.

Given the plethora of essential biological processes controlled by mTOR, it is not surprising that mTOR-signaling dysfunctions have been reported in many pathological conditions including cancer, metabolic disorders, and neuromuscular and neurological diseases [3]. Consequently, this signaling hub and its multiple routes of regulation constitute putative therapeutic targets [15]. Interestingly, treatment with rapamycin, a specific inhibitor of mTORC1, increases lifespan in several species showing that mTOR-signaling regulation is also crucial during aging [16]. However, much remains to be known about mTOR signaling and developing new tools will help to unveil how activation and regulation of mTOR would impact cell growth and metabolism in normal and pathological conditions.

Quantification of the phosphorylation of specific mTOR substrates by Western blotting is the most widely used method to monitor mTOR activity. Whereas it is efficient to picture an endpoint readout of mTOR activity, this approach requires nevertheless the use of cellular lysates and is time consuming and relatively costly. Besides, it is hard to reach a consensus for the analysis of mTORC1 activity because it has many different targets which may not be activated altogether despite mTORC1 being indeed activated. In any case, this biochemical approach is unable to reveal dynamic or subtle changes of mTOR activity in living cells upon various conditions.

Efforts have been made to generate biosensors able to detect kinase activity in living cells [17, 18] mostly based on the FRET approach (fluorescence resonance energy transfer). These genetically encoded intramolecular FRET biosensors monitor conformational change-induced proximity between donor and acceptor fluorophores upon phosphorylation of a specific kinase substrate. An mTOR FRET biosensor containing the 4EBP1 substrate was recently described, which is able to measure mTOR activity by FRET in cell imaging experiment [14]. However, this biosensor has been validated in experimental stimulation/inhibition of mTOR activity, but not tested yet upon native activity changes in physio-pathological conditions. This highlights the difficulty and need to develop a highly sensitive biosensor.

Bioluminescent Resonance Energy Transfer (BRET)-based biosensors display high sensitivity, thanks to the excellent signal-to-noise ratio of the BRET approach [19–21]. Using a small and bright nanoluciferase [22, 23], we further improved the sensitivity of BRET imaging [21]. These recent developments make BRET technology a well-suited and sensitive method for biosensor design and measurement in cell population studies or cellular imaging in living cells [24].

In this study, we designed an mTOR BRET biosensor and verified its specificity and response towards negative and positive mTOR cues. We then used this biosensor to investigate mTOR activity in two relevant cellular contexts: (i) Using versions of the biosensor targeted to various subcellular compartments, we evidenced an increase of mTOR signaling and the existence of discrete subcellular pools of mTOR activity during the course of muscle cell differentiation and in response to leucine stimulation. (ii) We depicted in real time the kinetics of depolarization-induced mTOR activation in living neurons from wild type mice and mTOR-signaling dysfunctions in neurons from *Fmr1* KO mice and Shank3Δ11 mice, two mouse models of intellectual disability (ID) and autism spectrum disorder (ASD).

## Material and methods

### AIMTOR biosensor plasmid constructs

The AIMTOR biosensor was derived from the YEN ERK biosensor plasmid described in Goyet et al. [21]. The sequence encoding the ERK-phosphorylated peptide in this biosensor was removed by BspEI/NotI double digest and replaced by PCR products encoding for human ULK1 peptide and mutant (S757/T757/A757) or human 4EBP1 peptides (S65T70, T37T46, aas [1–51], and full). The cytosolic biosensor harbors a C-terminus nuclear export signal (NES) right after the nanoluciferase sequence maintaining AIMTOR in the cytosol. To make nuclear, mitochondrial, or lysosomal targeted biosensors, this NES sequence was removed by XbaI/SalI digestion and ligated with complementary oligonucleotides encoding either a C-terminus SV40 nuclear localization signal (NLS), a mitochondrial (MITO) targeting sequence from the human monoamine oxidase (last 29 aa C-terminus sequence) [25], or a 15 aas C-terminus CAAX lysosomal targeting sequence from the Rheb protein [26]. The plasmids encoding for AIMTOR and its non-responding mutant are available at Addgene (AIMTOR Addgene ID: 140828 and non-responding mutant AIMTOR T757A Addgene ID: 140829). For neuronal transduction, the AIMTOR coding sequence was sub-cloned in an AAV backbone containing the hSynapsin promoter. All constructs were verified by sequencing after each cloning step.

### Cell culture

C2C12, H1299, and HEK293T were cultivated in DMEM 4.5 g/l glucose (Sigma D5796) supplemented with 10% vol/vol of fetal bovine serum, glutamine 2 mM, pyruvate 1 mM, and antibiotics (penicillin/streptomycin). However, for BRET reading, biosensor expressing cells were grown, split, or seeded in DMEM medium depleted in phenol red to avoid interference with BRET measurement (see the “BRET measurement” section).

For differentiation experiments with C2C12 cells, 24 h after transfection of the biosensor, cells were split in 96 Well clear bottom microplates (Greiner) for BRET cell population measurement or in 4 or 8 multiwell slides (IBIDI, CliniSciences, France) for BRET imaging or confocal fluorescent imaging. The proliferation medium containing 10% of serum was replaced by the differentiation medium containing 0.5% or 2% serum. Cells were fed every other day with fresh medium, and the medium was also changed immediately before any BRET measurement.

### Primary muscle cells

Quadriceps muscle biopsy was from one healthy adult and was done at the Centre Hospitalier Universitaire Lapeyronie (Montpellier, France). All volunteers signed an informed written consent after description of the protocol (authorization no. DC-2008-594). Myoblasts were purified from the muscle biopsy and were cultured on collagen-coated dishes in DMEM/F12 medium with 10% fetal bovine serum (FBS), 0.1% Ultroser G, and 1 ng/ml of human basic fibroblast growth factor (proliferation medium), as previously described [27].

### Mice

*Shank3Δ11* and *Fmr1* mice were previously described in [28] and [29], respectively. They were housed under constant temperature (22 ± 1 °C) and humidity (50%) conditions with a 12-h light/dark cycle and provided with food and water ad libitum. Using heterozygous mice for breeding, we derived wild type and knockout littermates.

### Hippocampal neuronal cultures

Cultures were prepared as recently described [30]. Briefly, hippocampi from P0–P3 mice were dissected and grown in neurobasal-A medium supplemented with B27 2% (Gibco), Glutamax 0.25% (Gibco), l-glutamine 0.5 mM (Gibco), fetal bovine serum 10% (Gibco), and antibiotics (penicillin and streptomycin) 1% (Gibco). After 2 days, culture was supplemented overnight with Cytosine β-D-arabinofuranoside hydrochloride (Sigma-Aldrich) 1 μM to curb glia proliferation. Then, approximately 75% of the culture medium was replaced by BrainPhys medium (Stemcell Technologies) supplemented with B27 2% (Gibco), Glutamax 0.25% (Gibco), and antibiotics (penicillin and streptomycin) 1% (Gibco).

### Cell transfection and transduction

#### Transfection

Cells were seeded in six-well plates (well diameter 35 mm) at a density of 150K cells/well (H1299 and C2C12) or 300K cells/well (HEK293T) 1 day before transfection. Transfections were carried out with 1 μg/well of DNA including 5 to 15 ng of the mTOR biosensor plasmid for transfection in H1299 and HEK293T cell lines or 50 to 100 ng of biosensor plasmid for transfection in C2C12 or in human primary cells. A “pBluescript” non-coding plasmid was used to normalize DNA amount to 1 μg. We routinely transfect cells using JETPEI (Polyplus, France) according to the manufacturer recommendations (4 μl/μg of DNA). According to the cell type and the transfection efficiency, it is important to optimize the amount of biosensor plasmid transfected to limit its expression in order to maximize its responsiveness and/or its dynamic range.

#### Transduction

AAV-hSynapsin-T757 AIMTOR particles were prepared using the ViraBind AAV Purification Kit (Clinisciences) according to the manufacturer protocol. After 6 days in culture, neurons were transduced with AAV-hSynapsin-T757 AIMTOR particles and in vitro BRET experiments were performed from days 13 to 15.

### Reagents

Rapamycin and Torin 1 were purchased from Calbiochem (Merck) and resuspended in DMSO. Phosphatase inhibitors cocktail 3 (P0044) is from SIGMA. Furimazine was purchased from Promega (France).

### Amino acid stimulation experiments

The DMEM/HAM F12 cell culture medium (Sigma D9785) without K, L, M, and Q and without phenol red was supplemented with 10% FCS serum, 1/100 penicillin/streptomycin, and as needed with methionine, lysine, leucine, and glutamine solution. It is to note that amino acid stimulation of mTORC1 required serum to release the TSC1/TSC2 brake, which prevents mTORC1 pre-activation by Rheb. Therefore, to remove all putative trace of amino acid present in the serum that could interfere with the efficiency of the amino acid deprivation step, two 16-h-long dialysis steps were carried out at 4 °C in 2.5 l of PBS for 50 ml of serum and the resulting dialyzed serum was filtered-sterilized. Then, AIMTOR biosensor transfected differentiated C2C12 or human primary myoblasts grown in rich medium were rinsed twice in PBS and incubated with medium containing dialyzed serum and lacking K, L, M, and Q during 3 h. This amino acid-deprived medium was replaced by either rich medium or medium supplemented with only leucine or medium supplemented with leucine and rapamycin (250 nM). All media were changed to fresh ones immediately before BRET readings.

### siRNA silencing

REDD1 (siREDD1) and negative control (siCTRL) Silencer Select pre-designed siRNAs were purchased from Life Technologies (France). H1299 cells were transfected with siREDD1 (#S29168) or a control siRNA (Ambion) as previously described [31].

### BRET measurement

BRET measurement in cell lines and primary muscle culture were carried out in DMEM (Life Technology, ref A14430) containing no phenol red to prevent interferences with BRET measurement and supplemented with serum, glutamine, pyruvate, and 25 mM glucose. Hepes 15 mM pH 7.4 was added to the medium in BRET imaging experiments to prevent pH drop during BRET imaging. BRET measurement in neurons was performed in artificial cerebrospinal fluid (ACSF): NaCl 140 mM, CaCl_2_ 2 mM, KCl 3 mM, Hepes 10 mM, glucose 10 mM, tetrodotoxin 0.0003 mM, glycine 0.01 mM, and bicuculline 0.01 mM, pH 7.4 with an osmolarity around 320 mOsm. In neurons, BRET recordings were started 45 min after media change. The basal mTOR signal recorded in ACSF control condition slightly decreased linearly over time. This linear regression has been modeled (GraphPad Prism 7.05) and the fit subtracted to BRET measurements displayed in Fig. 6a.

### BRET measurement in cell population

To measure BRET signal in 96-well white microplate, the medium was changed with fresh one immediately before BRET reading with 90 μl of medium/well. Furimazine (Promega, France) diluted in the medium was added to each well at 20 μM. BRET signal was then measured in triplicate wells over a 10-, 20-, or 60-min period depending of the experiment, with a Biotek synergy2 reader that allows to sequentially detect the light emission signal at 530 nm and 480 nm (1-s reading for each wavelength). The BRET signal was then calculated by determining the emission ratio 530nm/480nm and in some experiments subtracting the basal BRET value from non-stimulated cells or fasting conditions.

### BRET imaging

Single-cell BRET imaging experiments were performed as previously described [21]. Briefly, the 535nm/450nm ratio signals were recorded using a bioluminescence-dedicated inverted fluorescence microscope (Axiovert 200M; Carl Zeiss) with a Plan Apochromat × 40 or × 63/1.40 oil M27 objective at room temperature and collected with an Evolve camera (Photometrics) equipped with an EMCCD detector, back-illuminated, On-chip Multiplication Gain. Sequential acquisitions of light emission at 450-nm and 535-nm wavelengths (Em450 and Em535) were performed at 5 MHz, gain 200, binning 1, with emission filters 450/70 nm (FF01-450/70-25, Semrock) and HQ535/50 nm (No. 63944, Chroma), respectively. Furimazine (50 μM) was applied 2 min before acquisition using MetaMorph software (Molecular Devices). Images were analyzed and BRET ratios calculated with “BRET analyzer,” an open-source toolset for Fiji [34].

### Confocal imaging

C2C12 myotubes expressing a cytosolic (NES), nuclear (NLS), mitochondrial (MITO), or lysosomal (LYSO) AIMTOR were imaged with a confocal microscopic device ZEISS LSM880 on the MRI-DBS facility (University of Montpellier). Images represent optical slices taken on a × 40/1.4 Plan Apochromat objective, with a Fast-airyscan process in a super-resolution mode (increases the confocal resolution by 2). Lasers used were 488 nm for YPET and 561 nm for mitotracker and lysotracker.

### Western blot analysis

Western blots were performed as previously described [35]. Briefly, the following antibodies were incubated on membranes overnight: anti-P-S6K1 (1/1000) (Cell Signaling Technology # 9206,) anti-Myogenin (1/100) (Santa-Cruz, sc-576), and anti-REDD1 (1/1000) (ProteinTech 10638-1-AP). The next day, membranes were washed and incubated with horseradish peroxidase (HRP)-conjugated secondary antibody at 1/3000 for 1 h at room temperature. Membranes were developed with an enhanced chemiluminescent (ECL) reagent from Biorad using a Chemidoc™ Touch Imaging System (BioRad). The stain-free system from Biorad was used to quantify the total protein load and to normalize phosphorylated S6K1.

### Statistics

Values shown are mean + SEM of at least 3 independent transfections, each measured in triplicate. Statistics were analyzed by using GraphPad Prism software. Statistical differences were analyzed by a two-tailed Student *t* test (to compare two conditions) or one-way ANOVA (to compare more than two conditions) using a parametric or non-parametric test, depending on the sample size. When significant, post hoc multiple comparison test was carried out. In some experiments, grouped two-way ANOVA analysis was used as mentioned in the figure legend. For BRET imaging studies, the non-parametric Mann and Whitney test was used to compare two independent samples: *p, 0.05; **p, 0.01; ***p, 0.001; ****p, 0.0001.

## Results

### mTOR biosensor design and validation

**Fig. 1:**
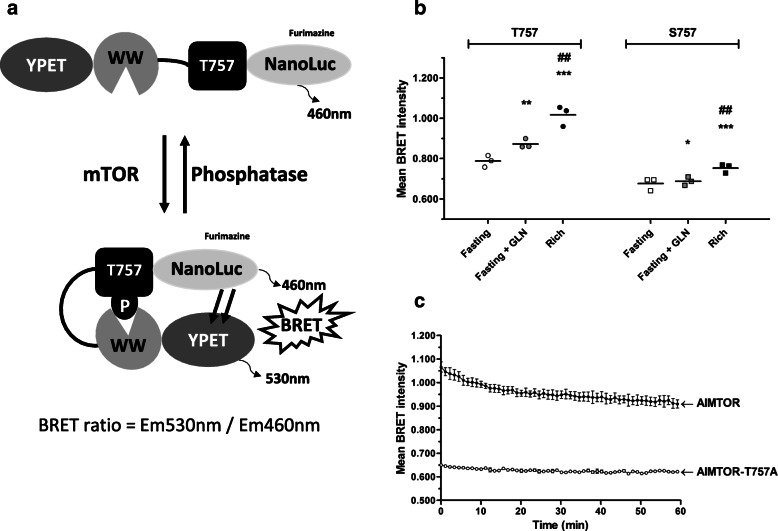
mTOR biosensor design and peptide selection. **a** Schematic representations of the conformational changes of AIMTOR induced upon mTOR activation (adapted from [19]). The biosensor contains a phosphorylable T757 peptide derived from the mTOR substrate, ULK1, a flexible linker and a WW domain able to recognize phosphorylated serine or threonine residues. These elements lay between a nanoluciferase donor entity emitting light at 460 nm after furimazine addition and a YPET acceptor protein emitting light at 530 nm. Upon mTOR phosphorylation, the WW domain will bind to the phosphorylated peptide increasing the proximity between nanoluciferase and YPET, which results in a BRET increase measured in live cells using a luminometer or a microscopic device (BRET ratio = Em530nm/Em460nm). **b** mTOR biosensor optimization. BRET measurements in live H1299 adherent cell population transfected with ULK1 peptides (S757 or T757)-containing biosensors. Cells were incubated in different media: “Fasting” medium (devoid of glutamine and serum), “Fasting + GLN” (fasting medium + glutamine), and “rich” medium (supplemented with glutamine and 10% serum). Each dot on the graph represents the mean of the BRET intensity recorded over 20 min for T757 (circles) and S757 (squared) expressing cells, respectively. The horizontal black bar shows the mean of BRET intensities of 3 independent experiments. One-way ANOVA with post hoc using the Tukey multiple comparison test. *p < 0.05, **p < 0.01, and ***p < 0.001 compared to the “Fasting” condition. ^##^*p* < 0.01 compared to the “Fasting + GLN” condition. **c** Kinetics of net BRET intensity in H1299 suspension live cells transfected with AIMTOR or AIMTOR-T757A mutant plasmids. Mutating the mTOR-targeted threonine residue in AIMTOR into a non-phosphorylable alanine strongly reduces BRET intensity

To design a biosensor specific to the activation of the mTOR kinase, we used a phosphorylable sensor peptide derived from a natural mTORC1 substrate (Fig. 1a, Additional file 1: Fig. S1A and S1B). Near this specific sensor region lies a flexible linker and a WW ligand-binding domain able to recognize and bind phosphorylated-serine/-threonine residues. The biosensor was flanked by the nanoluciferase as energy donor at the C-terminus position and the YPET fluorescent acceptor located at the N-terminus (Fig. 1a). This BRET donor/acceptor combination is known to strongly increase the sensitivity of BRET biosensors [21]. Upon mTOR phosphorylation of the substrate peptide, the binding of the WW domain to the phosphorylated residue induces a conformational change of the biosensor, which increases proximity between donor and acceptor entities resulting in BRET enhancement (Fig. 1a).

We selected among several natural substrates of mTORC1 the best one, giving the highest BRET dynamic range response (Additional file 1: Fig. S1B and S1C). All these peptides have been shown to be efficiently phosphorylated by mTORC1 [36, 37]. mTOR biosensor selection was performed measuring BRET intensities in fasting (without serum and glutamine) and rich media conditions, predicted to give rise to a low and high mTOR biosensor BRET activity, respectively. The best responding BRET biosensor contained a short peptide derived from the ULK1 protein encompassing the Serine 757 phosphorylable by mTORC1 (Additional file 1: Fig. S1A, S1B, S1C).

Other mTORC1 targets like full-length 4EBP1 or peptides containing known mTORC1 phosphorylation sites of 4EBP1 (T37-T46 or S65-T70) were either inefficient or less active in this BRET assay compared to ULK1-containing mTOR Biosensors (Additional file 1: Fig. S1C). Published data from Kang and colleagues indicated that replacing the serine by a threonine in several mTORC1 substrates modified their properties as mTOR substrates [37]. Therefore, to optimize the biosensor, we mutated the native phosphorylable serine 757 of the ULK1 substrate sequence into a threonine residue (T757). Interestingly, this T757 biosensor configuration exhibited a better BRET response than the native ULK1 sequence (Fig. 1b). Importantly, the BRET difference between the rich and fasting condition reached ~ 0.230 BRET units for the T757 biosensor version compared to ~ 0.076 BRET units for the S757 biosensor. Moreover, a subtle stimulation like glutamine-induced mTOR activation was easily detected with the T757 biosensor (+ ~ 0.085 BRET units), whereas it was hardly detected with the S757 biosensor (+ ~ 0.011 BRET units, Fig. 1b). This increased dynamic window for BRET variations prompted us to select the ULK1-derived T757 biosensor as efficient mTOR activity reporter, which we named AIMTOR. As an important control, we built an AIMTOR-T757A mutant biosensor harboring a non-phosphorylable alanine in place of the phosphorylable threonine residue in the ULK1 peptide. This non-responding mutant exhibited only basal BRET activity as expected (Fig. 1c).

### AIMTOR reports mTOR-triggered phosphorylations

We further characterized AIMTOR responses to mTOR-specific inhibitors and several pertinent cues known to activate the mTOR-signaling pathway.

**Fig. 2:**
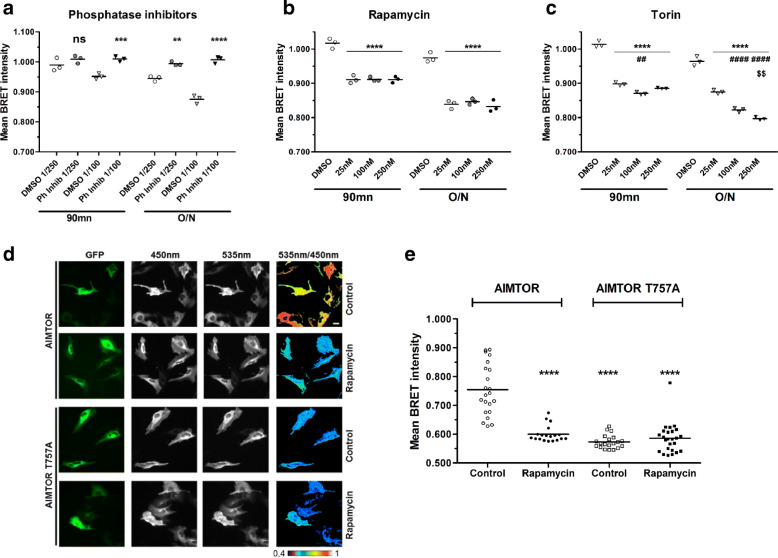
AIMTOR responses to phosphatase and mTOR inhibitors. **a**–**c** AIMTOR reports phosphorylation-dependent events, triggered by mTOR. Mean BRET intensities recorded in H1299 cells expressing AIMTOR. Cells were incubated for 90 min or overnight (O/N) either with a mix of serine/threonine phosphatase inhibitors (**a**), the mTOR inhibitor rapamycin (**b**), and Torin (**c**) or with the DMSO vehicle as control. Each dot on the graphs represents the BRET intensity of one experiment and the horizontal bar is the mean of BRET intensities recorded in 3 independent experiments. Grouped analysis, 2-way ANOVA (“time” and “treatment”) with post hoc using the Bonferroni multiple comparison test. **p < 0.01, ***p < 0.001, and ****p < 0.0001 compared to the respective DMSO (control) condition. ^##^*p* < 0.01 and ^####^*p* < 0.0001 compared to the 25 nM condition. ^$$^*p* < 0.01 compared to the 100-nM condition. The interaction between “time” and “treatment” is significant in **a** (*p* = 0.0006) and non-significant in **b** (*p* = 0.0959) and significant in **c** (*p* < 0.0001). **d**, **e** BRET imaging of mTORC1 activities detected with AIMTOR. Biosensor-transfected C2C12 cells were treated overnight with rapamycin (250 nM) or DMSO (control). Reporter expression is reported by YPET expression (GFP) and NLuc emission (Em450), whereas the excitation of YPET by the non-radiative transfer of energy from NLuc is recorded in the Em535 image. Pixel-by-pixel division of the Em 535 and Em 450 images gives rise to the pseudo-colored BRET image (Em 535/Em 450). Representative images (**d**) and quantification of the average 535nm/450nm ratio intensities (**e**) of AIMTOR and mutant AIMTOR-T757A signals in transfected proliferating C2C12 myoblasts. Each dot in the graph represents the BRET intensity recording of one cell, and the horizontal bar shows the mean obtained from 20 to 25 cells per condition. Mann-Whitney statistical analysis with ****p < 0.0001 compared to the AIMTOR control condition

We first controlled that AIMTOR BRET intensity significantly rose in the presence of phosphatase inhibitors (Fig. 2a), indicating that phosphorylation/dephosphorylation events triggered AIMTOR BRET changes.

As expected, AIMTOR responded to incubation with mTOR inhibitors such as rapamycin, considered as an mTORC1-specific inhibitor, and Torin1, a generic mTOR inhibitor (Fig. 2b, c, and Additional file 2: Fig. S2A). Of note, a significant BRET decrease was detected with a low concentration of the drugs (25 nM) and with the shorter incubation time, indicating the good sensitivity of AIMTOR (Fig. 2b, c). Moreover, we observed a dose effect of Torin after O/N incubation suggesting that at high concentration, Torin could inhibit several complexes containing mTOR with an enhanced negative impact on AIMTOR BRET intensity (Fig. 2c). We also reported that a biosensor containing the native S757 ULK1 peptide responded as well to mTOR inhibition (Additional file 2: Fig. S2A) although again with a reduced dynamic range and sensitivity compared to AIMTOR (− ~ 0.05 BRET units for S757 versus − ~ 0.1 BRET units for AIMTOR in a 90 mn Torin incubation).

In BRET imaging experiments, AIMTOR displayed non-uniform BRET intensities among cells, which were once again decreased by rapamycin application. By opposition, the AIMTOR-T757A mutant exhibited a uniform weak basal BRET, insensitive to mTOR inhibitors (Fig. 2d, e). Interestingly, those cell-to-cell variations of AIMTOR BRET intensities are likely to disclose a fine-tuning of mTOR signaling between cells which could not have been evidenced by other methods (Fig. 2d). Comforting this, we did not find any correlation between the level of expression of AIMTOR and its BRET intensity (*R*^2^ = 0.02207 for linear regression fit) (Additional file 2: Fig. S2B). Therefore, it is reasonable to propose that the changes of BRET intensity observed among individual cells are due to actual biological relevant variation(s) of mTOR activity. Altogether, these data indicate that AIMTOR up and down BRET changes measured in various conditions depend on direct phosphorylation of the target peptide by mTOR.

**Fig. 3:**
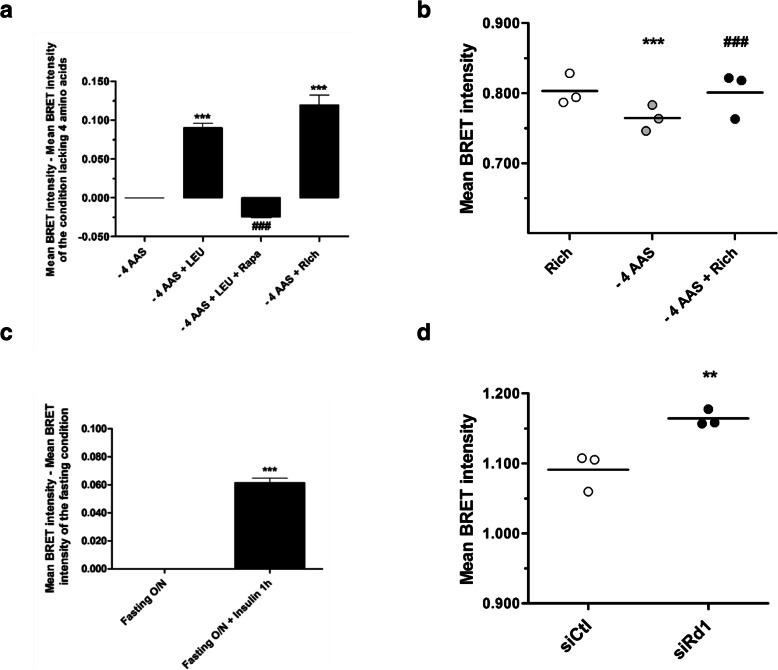
AIMTOR responses to specific mTOR signaling cues. **a**, **b** AIMTOR BRET intensity increases in response to amino acid stimulation. **a** AIMTOR BRET intensities in C2C12 cells fasted 3 h in medium lacking four amino acids (leucine, methionine, lysine, and glutamine) then incubated 1 h with the same medium, or medium supplemented with leucine, rapamycin, or with leucine, methionine, lysine, and glutamine (“- 4 AAs + Rich”). Histogram: mean ± SEM of BRET intensity, with BRET intensity from “- 4AAs” condition subtracted from the indicated condition. *n* = 3–6 independent experiments. One-way ANOVA repeated measures, post hoc analysis with Tukey’s multiple comparison test: ***p < 0.001 compared to “- 4 AAs” condition, ^###^*p* < 0.001 compared to “- 4 AAs + Leucine” condition. **b** AIMTOR BRET intensities in human primary muscle cells incubated in “rich” medium (white circles) or fasted 3 h in medium lacking four amino acids and then treated for 1 h in the same medium (gray circles) or in rich medium (black circles). Dots represent single experiments; horizontal bar shows the mean of 3 independent experiments. One-way ANOVA repeated measures, post hoc analysis with Tukey’s multiple comparison test: ***p < 0.001 compared to “rich “condition and ^*###*^*p* < 0.001 compared to “- 4 AAs” condition. **c** Insulin increases AIMTOR BRET intensity: mean ± SEM of BRET intensity of AIMTOR-transfected C2C12 cell serum fasted overnight and stimulated or not with insulin for 1 h (*n* = 6), with BRET intensity from the “O/N serum fasted” condition subtracted from each condition. Mann-Whitney *t* test, ***p < 0.001. **d** Silencing of REDD1, a negative regulator of mTORC1, increases AIMTOR BRET intensity. Dots represent single experiments of H1299 cells expressing AIMTOR previously transfected with a siRNA targeting REDD1 (black colored circles) or a non-targeting control siRNA (white colored circles). Horizontal bar: mean BRET intensity of 3 independent transfections. Wilcoxon two-tailed matched paired sign rank test, **p < 0.01

We then carried out experiments to characterize further the AIMTOR BRET intensity in response to specific cues known to affect mTOR signaling. AIMTOR intensity decreased in response to amino acid depletion and increased in the presence of leucine and other amino acids in differentiated C2C12 myotubes or in primary myoblasts purified from a human muscle biopsy (Fig. 3a, b). Moreover, the leucine-mediated increase of AIMTOR BRET intensity was abolished in the presence of rapamycin (Fig. 3a). AIMTOR was also sensitive to insulin stimulation, a well-known mTORC1 activator (Fig. 3c). Lastly, when the expression of REDD1, a negative regulator of mTORC1, was reduced by siRNA silencing, AIMTOR BRET intensity significantly increased and was accompanied by a rise in the phosphorylation of S6K1 as assessed by Western blot (Fig. 3d and Additional file 2: Fig. S2C). Therefore, AIMTOR signal is sensitive to specific cues activating mTORC1.

**Fig. 4:**
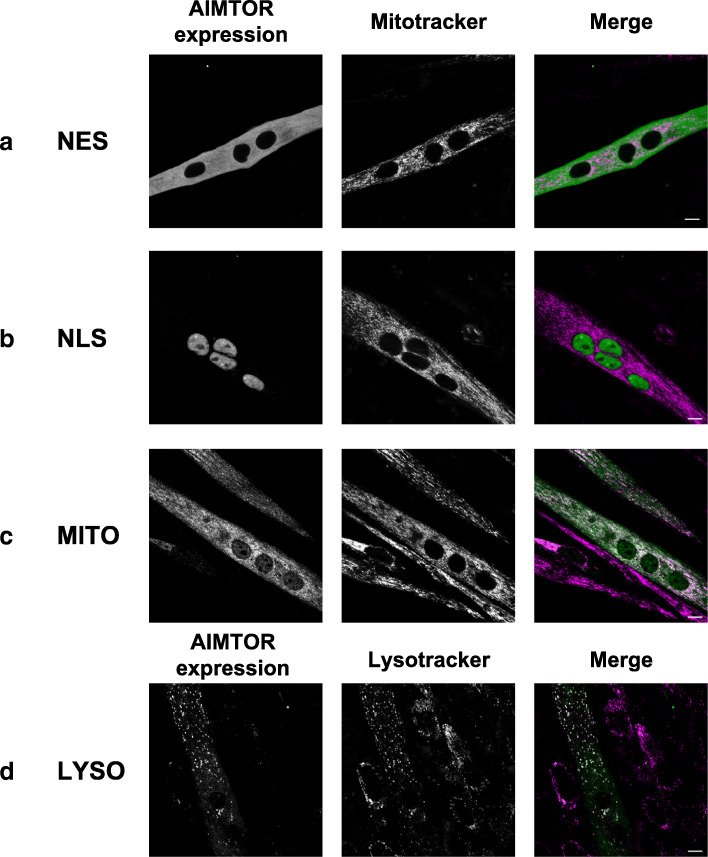
Targeting AIMTOR to various subcellular localizations. **a**–**d** Fluorescence microscopy of living differentiated C2C12 myotubes expressing either cytosolic (NES) (**a**), nuclear (NLS) (**b**), mitochondrial (MITO) (**c**), or lysosomal (LYSO) AIMTOR (**d**). The left and middle channels show in a gray scale LUT YPET fluorescence of AIMTOR and either mitochondria (**a**–**c**) (labeled with mitotracker) or lysosomes (labeled with lysotracker Red99) (**d**), respectively. For the merge image on the right, the YPET fluorescence of AIMTOR is in green and mitochondria or lysosomes are in magenta. Scale bar represents 10 μm

MTORC1 activation occurs mainly at the surface of lysosomes and presumably at several other subcellular localizations. To test this hypothesis, we built different variants of AIMTOR containing specific C-terminus localization signal cassettes, to monitor mTORC1 activity in a single specific subcellular compartment. We performed live myotube confocal imaging to confirm that the four versions of AIMTOR were indeed targeted to their expected localization (Fig. 4). Besides our reference biosensor containing a C-terminus NES (nuclear export signal) and localized in the cytosol (Fig. 4a), we targeted AIMTOR into the nucleus (Fig. 4b), to the external membrane of the mitochondria (Fig. 4c), or to the lysosome surface (Fig. 4d).

We then used AIMTOR to report mTOR activities in physiological conditions in muscle cells and in neurons.

### AIMTOR intensity increases during muscle cell differentiation

**Fig. 5:**
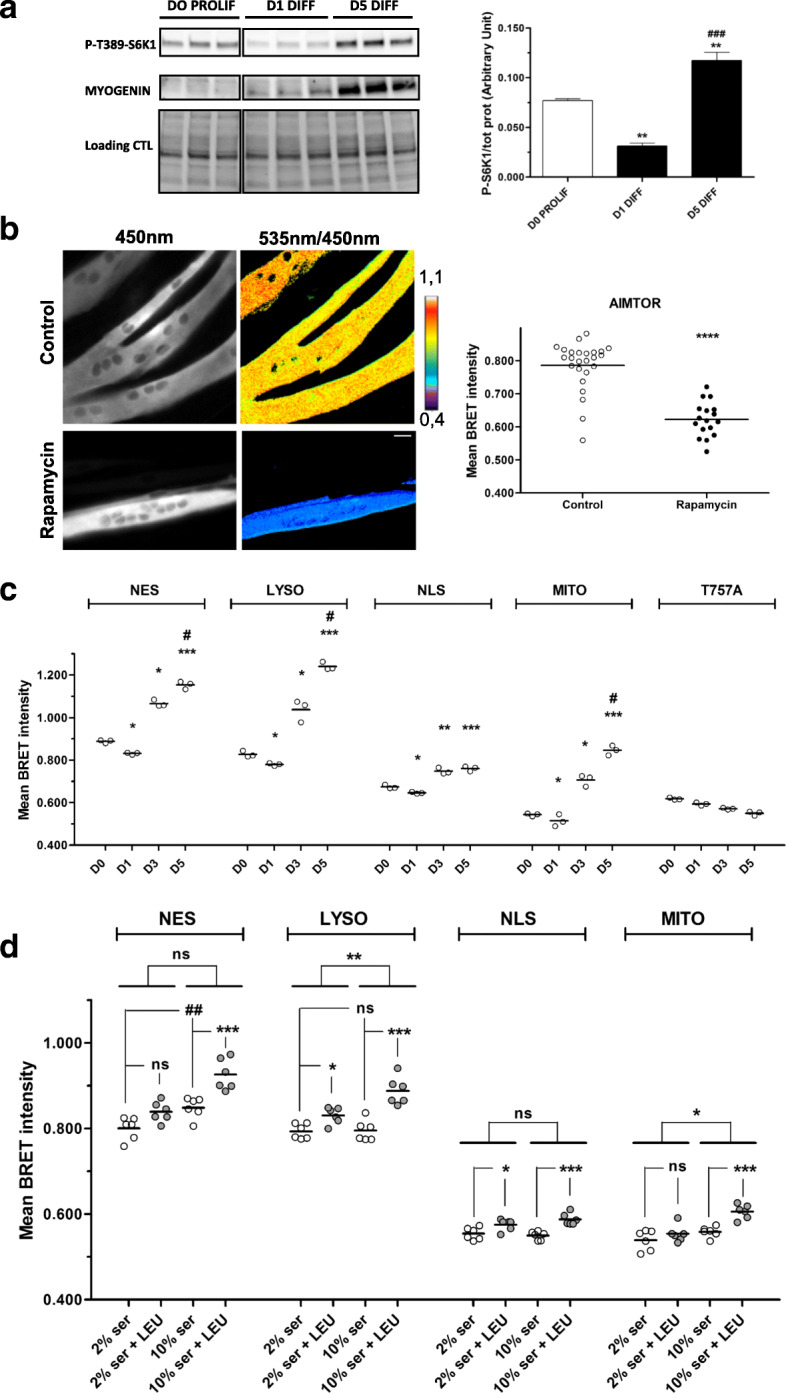
AIMTOR reports mTORC1 activity in differentiating muscle cells. **a** mTORC1 activity in C2C12 differentiating cells (triplicates wells for each stage). Bands show phosphorylated S6K1 (above), Myogenin, a differentiation marker (middle) and total protein load revealed (below). Right: quantification of phosphorylated S6K1 bands normalized by total protein load. One-way ANOVA, multiple comparison Tukey’s post hoc test. **p < 0.01 compared to D0 and ^###^*p* < 0.001 compared to D1. **b** BRET imaging of 5-day differentiated C2C12 expressing cytosolic AIMTOR treated overnight with rapamycin or DMSO (control). Right: BRET intensity of individual cells treated with DMSO or rapamycin; horizontal bars show mean BRET intensity calculated from 20 to 25 cells per condition. Mann-Whitney statistical analysis with ****p < 0.0001 compared to control condition. **c**, **d** Mean BRET intensity of differentiating C2C12 cells expressing AIMTOR localized to various subcellular compartments: each dot represents the mean BRET intensity of one experiment. In **c**, BRET intensity recorded in cells in their proliferative phase (D0), at day 1 (D1), day 3 (D3), and day 5 (D5) of differentiation and expressing, cytosolic (NES), lysosomal, nuclear, mitochondrial AIMTOR or the T757A non-responding mutant. One-way ANOVA non-parametric Friedman test with repeated measure and multiple comparison Dunn’s post hoc test. *p < 0.05, **p < 0.01, and ***p < 0.001 and compared to D0. ^#^*p* < 0.05 compared to D3. In **d**, BRET intensity in 5-day differentiated C2C12, fasted with medium lacking four amino acids containing 2% or 10% of dialyzed serum, and stimulated (gray circles) or not (white circles) with leucine during 1 h. Grouped analysis, two-way ANOVA (“leucine treatment” and “serum concentration”) with post hoc using the Bonferroni multiple comparison test. *p < 0.05 and ***p < 0.001 compared to the fasting condition. Grouped analysis also performed for cytosolic and lysosomal AIMTOR, two-way ANOVA (“Biosensor Localization” and “serum concentration”). The interaction is significant (*p* = 0.0269). ^##^*p* < 0.01 compared to the 2% serum condition

During myoblast differentiation into myotubes, mTORC1 activation rises, paralleling protein accretion to sustain the differentiation program and cell growth [38, 39]. We confirmed this during C2C12 muscle cell differentiation by Western blot analysis based on S6K1 phosphorylation quantification (Fig. 5a). We then used AIMTOR to refine over time the mTOR activity profile in C2C12 differentiating cells. AIMTOR BRET intensity increased at days 3 and 5 of differentiation when myoblasts have fused to form myotubes (Fig. 5b, c). The slight decrease of AIMTOR BRET intensity measured early on at day 1 in myoblasts corresponds to the lowering of serum concentration of the culture medium to initiate the differentiation process (Fig. 5c). Importantly, the AIMTOR BRET intensity variations observed during C2C12 differentiation were specific of AIMTOR phosphorylation by mTORC1 since (i) low concentration of rapamycin abolished the AIMTOR BRET intensity increase observed at day5 (Additional file 3: Fig. S3A) and (ii) in comparison to AIMTOR, the AIMTOR-T757A mutant did not exhibit any BRET intensity increase during C2C12 differentiation (Fig. 5c and Additional file 3: Fig. S3B). Cytosolic and lysosomal AIMTOR exhibited a similar profile and range of BRET intensity variations during muscle cell differentiation (Fig. 5c). Besides, we detected mTORC1 activity into the nucleus and close to mitochondria with an increase at the end of the differentiation process of nuclear AIMTOR and mitochondrial AIMTOR BRET intensities, respectively (Fig. 5c). Rapamycin treatment confirmed the specificity of the BRET intensity increases recorded for all these AIMTOR targeted to various cell subcompartments assessed by BRET microscopy (Fig. 5b and Additional file 3: Fig. S3C, S3D and S3E) and in cell population (Additional file 3: Fig. S3F, S3G, S3H and S3I).

The surface of lysosome is a major site of activation for mTORC1 by amino acid although other subcellular sites of activation could also be important in this signaling [11, 14]. In differentiated muscle cells, not only lysosomal but also cytosolic, and to a lesser extent nuclear and mitochondrial AIMTOR BRET intensities, increased after leucine stimulation in the presence of serum (Fig. 5). Moreover, in leucine-depleted culture medium, only cytosolic AIMTOR BRET intensity rose with serum concentration whereas lysosomal AIMTOR BRET intensity remained unchanged (Fig. 5d). These results indicate that a cytosolic pool of mTORC1 is activated in the cytosol in response to serum independently of leucine, whereas on the surface of lysosome, mTORC1 activation required both leucine and serum. This shows that AIMTOR could help in understanding the subcellular compartments supporting mTORC1 activation by amino acid and other cues.

Collectively, our results establish the cytosol and the lysosome as major sites of mTORC1 activity in the establishment and/or maintenance of differentiated muscle cells and in amino acid signaling. However, AIMTOR also unravels discrete pools of mTORC1 activity in the nucleus and close to mitochondria that could be important in these processes.

### AIMTOR detects mTOR dysfunctions in autism spectrum disorders (ASD)

**Fig. 6:**
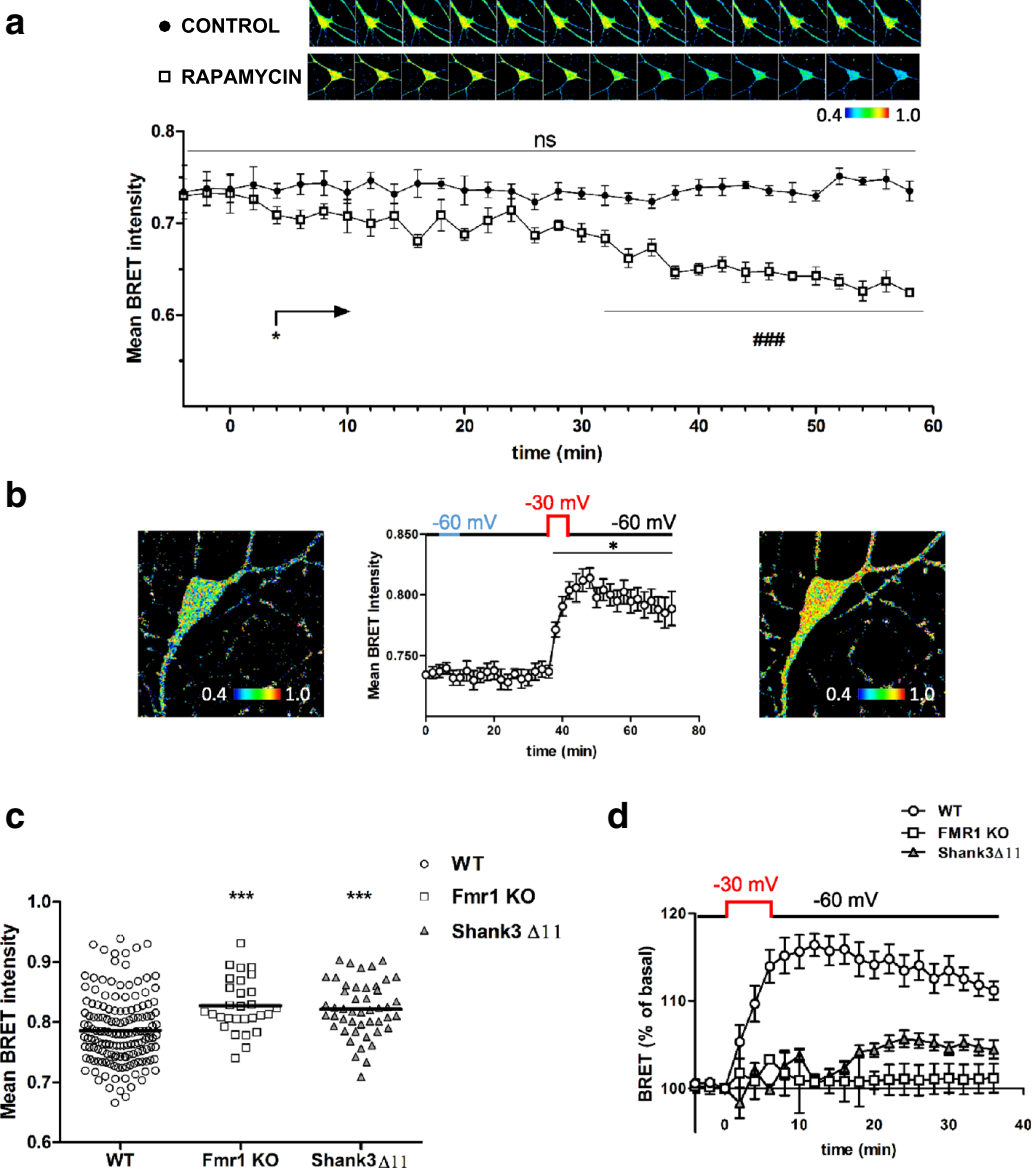
AIMTOR reports mTORC1 signaling intensities in primary cultures of hippocampal neurons. **a** Intensities of the 535nm/450nm ratio in AIMTOR transduced neurons recorded over time after DMSO (control) or rapamycin addition at *t* = 0. Representative images illustrate this ratio at the indicated times. **b** Mean BRET intensity recorded before and after transient perfusion of KCl 3 mM-containing ACSF (*t* = 4 to 10), followed by KCl 55 mM-containing ACSF (*t* = 36 to 42) to depolarize neurons from − 60 to − 30 mV. Representative images of *t* = 0 (left) and *t* = 58 min (right). **c**, **d** Basal 535nm/450nm ratio intensities (**c**) and depolarization-induced BRET increase expressed as percentage of the basal intensity (**d**), recorded in wild type (WT), Fmr1 KO, and Shank3Δ11 neurons. Each point represents the mean ± SEM for 16 to 30 neurons, 5 regions per neuron. One-way ANOVA multiple comparison statistical analysis for paired (**a**, **b**) and unpaired (**c**) data with *p < 0.05 and ***p < 0.001 compared to the control condition. In **a**, the initial one-way ANOVA multiple comparison analysis compared within each group the BRET intensity values at each time point to their respective basal BRET value (^###^*p* < 0.001). An additional test based on grouped analyses comparing each time point BRET intensity of the rapamycin versus corresponding DMSO control was carried out with a multiple *t* test where discovery is determined using the two-stage linear step-up procedure developed by Benjamini and colleagues [32, 33]. BRET intensity is different between rapamycin and DMSO conditions as early as 4 min (arrow *p < 0.05) after adding the drug and remains significantly below the DMSO condition (*p < 0.05, ***p < 0.001) until the end of the monitoring excepted for 3 measures (i.e., 10, 14, and 24 min)

The mTOR-signaling pathway is also a crucial hub in neurons, since it integrates cellular and extracellular signals to control fundamental cellular functions such as gene expression and metabolic balance ([40] for review). AIMTOR revealed a basal activity of mTOR signaling in control neurons, which was significantly reduced following rapamycin application (Fig. 6a). This basal activity of mTOR signaling could be significantly and stably exacerbated by a transient depolarization of neurons (Fig. 6b). The importance of mTOR signaling for brain function is highlighted by the numerous disorders in which mTOR pathway dysfunction is involved, like ASD [40]. However, real-time kinetics of mTOR activity were not reported yet because of the dearth of adequate technologies. Among mouse lines validated as ASD models, we worked with *Fmr1* knockout (KO) mice and Shank3Δ11 mice. The KO of *Fmr1* gene in mouse (*Fmr1*^−/y^) exhibits the primary molecular and behavioral symptoms associated with Fragile X syndrome (FXS), an inherited neurodevelopmental disorder with a wide variety of symptoms characteristic of ASD [41]. Shank3Δ11 mice display autistic-like behaviors and hyperactivity [28]. Interestingly, in neurons from *Fmr1* KO or Shank3Δ11 mice, AIMTOR reported a significantly higher basal activity of mTORC1 signaling compared to neurons from wild type mice (Fig. 6c). A transient neuronal depolarization failed to increase further mTOR signaling (Fig. 6d) in neurons from both ASD mouse models. Our data demonstrate that AIMTOR is an efficient sensor that can be used to phenotype mTOR signaling from primary neuronal cell culture of mice with CNS impairment associated with mTOR-signaling dysfunction.

## Discussion

In this study, we describe AIMTOR, a sensitive BRET biosensor allowing real-time cell measurements of mTORC1 signaling in cell population or in individual cells using BRET microscopy. We have achieved our goal to build a bona fide BRET mTOR biosensor that meets two important criteria: on one hand, this biosensor exhibits a wide dynamic range of BRET ratio changes allowing easy, sensitive, and gradual mTOR activity measurements. On the other hand, we validated the specificity of the biosensor with specific mTOR inhibitors (rapamycin and Torin 1) and stimulating cues (amino acids, insulin, and siREDD1).

### Biosensor peptide selection

During the initial biosensor optimization step, we selected the specific peptide region responding to phosphorylation by mTOR in reference to the study of Kang et al. who extensively studied mTOR substrate specificities and site position constraints [37].

We noticed that mutating the serine by a threonine residue in the original ULK1 peptide background provoked a marked amplification of the BRET changes without affecting mTOR biosensor specificity. Part of this signal amplification could originate from the differential ability of mTORC1 to phosphorylate threonine and serine residues as already described [37]. However, there are two groups of natural mTORC1 substrates, one with threonine residues phosphorylated by mTOR including usually poor mTORC1 substrates (more sensitive to rapamycin) and the other with phosphorylable serine that encompasses excellent mTORC1 substrates (more resistant to rapamycin). In contrast, our biosensors based on T and S757 ULK1-derived peptides did not exhibit any difference in sensitivity between rapamycin and Torin. The fact that the T757 and S757 peptides exhibited such a different range of BRET response may also depend upon the overall biosensor conformation and/or results from a better binding of the WW domain to a phospho-threonine than to a phospho-serine residue as observed by others (Watabe and Matsuda 2018, personal communication).

### AIMTOR biosensor records mTORC1 activity

Our data strongly support that the AIMTOR biosensor responded mainly to mTORC1 activation/inhibition: firstly, rapamycin (an inhibitor of FKB12 specifically targeting mTORC1) and Torin (an inhibitor targeting mTOR in mTORC1 and mTORC2 complexes) induced similar decrease of the biosensor BRET ratio. Secondly, several specific cues known to activate mTORC1 like amino acid serum or insulin stimulated the biosensor as well. Finally, silencing the mTORC1 negative regulator REDD1 had a positive effect on the BRET biosensor as expected. We cannot formally rule out that AIMTOR phosphorylation depends partly either on other kinases activity such as mTORC2 complex or on the recently discovered mTORC3 complex mTORC3 [42] or on a related kinase of the same PIKK family (DNAPK, ATM/ATR) or with overlapping sequence specificity. Future experiments will define the full spectrum of mTOR activity revealed by this biosensor.

### Sensitivity of AIMTOR to experimental conditions

The experimental conditions used to record mTOR activity with AIMTOR are important parameters to consider as mTOR responds to many nutriments present in the cell culture medium and potentially also during BRET recording. Interestingly, AIMTOR BRET intensity variations integrated the dual roles played by endogenous phosphatases and mTOR kinase. Therefore, the composition of the cell culture media and of the BRET monitoring media, the presence/absence of specific nutriments or phosphatase inhibitors, and potentially the variation of pH can affect the BRET intensity recorded with AIMTOR. Moreover, in the course of the experimental development of AIMTOR, we noticed that the cell type and the degree of cell confluence, using cells attached or in suspension, are issues that can all influence AIMTOR BRET intensity. Overall, the high sensitivity of AIMTOR makes it possible to apprehend a wide range of fine experimental conditions at the origin of variations in mTOR activation. However, using AIMTOR warrants careful control experiments and possibly, cell-specific optimization of conditions.

### AIMTOR is available in different flavors, depending on the subcellular compartment in which the activity is to be monitored

By adding specific C-terminus-encoded localization signals, AIMTOR can report subcellular pools of mTOR activity. We investigated that these derivatives of AIMTOR were indeed targeted to their expected localization. They all appear functional as their BRET ratio intensity varies upon incubation with rapamycin suggesting that AIMTOR subcellular targeting did not affect mTORC1 specificity recording. However, their minimum and maximum BRET ratios were not similar. Cytosolic and lysosomal AIMTOR reported high BRET ratio whereas nuclear and mitochondrial BRET ratio remained low in comparison. Conformational variations due to the C-terminus extension required for targeting or to some specific subcellular biochemical context may explain such min and max BRET intensity differences among targeted AIMTOR. In any case, the sensitivity of AIMTOR was sufficient to monitor mTOR activity at different subcellular localizations.

### mTOR signaling in differentiated muscle cells

We applied our BRET biosensor approach to investigate mTOR signaling and activity during muscle cell differentiation, and this brought interesting information that supports and extends previous published results [38, 39]. First, we confirmed that mTOR activation increased during the differentiation process. Interestingly, our results support the existence of different pools of mTOR activity during muscle differentiation that could contribute to the metabolic adaptation and proteostasis change occurring during this process.

However, by using differentially targeted biosensors, we pointed out that mTORC1 activity is high in the cytosolic and the lysosomal compartment in differentiated muscle cells and that both cytosolic and lysosomal AIMTOR BRET intensities increase in response to leucine stimulation. Interestingly, our results highlighted a cytosolic pool of mTORC1 activated by serum independently of leucine. Our data suggest some redistribution and/or variation of mTORC1 activity between the cytosol and the surface of lysosomes as supported by recently published observations deciphering Raptor dynamic lysosomal localization upon amino acid stimulation [43]. Therefore, our targeted AIMTOR can help to discriminate between lysosomal and cytosolic mTORC1 activities.

In addition, AIMTOR revealed mTORC1 activity within the nucleus notably increasing during muscle cell differentiation. Although still debated in the literature, mTOR had already been observed by others into the nucleus of muscle cells where it could contribute to gene transcription regulation, metabolic adaptation, and reprograming occurring during differentiation [44]. Here we further showed that nuclear AIMTOR BRET intensity increased after leucine stimulation comforting the existence of an amino acid-dependent nuclear mTORC1 signaling as assessed by Zhou and colleagues [14].

Concerning mitochondria, whereas different authors have found mTOR associated with mitochondria, and in mitochondrial-associated ER membranes (MAM), few studies have investigated what role mTOR activity could have at this localization [45, 46]. As the mitochondria network undergoes an important remodeling during myotube differentiation [47, 48], one hypothesis is that the parallel increase of mTOR activity observed here proximal to mitochondria reflects the coordinated rise of protein translation, a major energy-consuming process, with ATP production by mitochondria. Our results also suggest that a mTORC1 pool localized near the mitochondria could be important in orchestrating cell signaling in response to leucine stimulation.

In addition, several organelle or membrane compartments interact in specific zones such as MAM, and recently, lysosome and mitochondria have been shown to be intertwined [49]. As mTOR can be a resident protein in these interaction zones, studying its role and activity warrant further investigations notably upon amino acid stimulation.

Extending previous observations, our targeted AIMTOR are therefore potent tools to measure discrete pools of mTORC1 activity in cells and to dissect their respective kinetics and contribution to shape an integrated mTOR signaling output [14].

### mTOR signaling in nerve cells

mTOR is known to play a key role in neuronal plasticity [50]. Using AIMTOR, we here disclose the state of mTORC1 substrate phosphorylation in neurons. Our data showed that in physiological conditions mTOR basal signaling is regulated by KCl-induced neuronal stimulation. The mTOR pathway in neurons is activated by glutamatergic stimulation. Previous reports described mTOR activation in a calcium/calmodulin-dependent manner through a calcium pool controlled by postsynaptic voltage-dependent calcium channels [51, 52]. Gq-coupled neurotransmitter receptor activation, like the mGlu5 receptor, can also activate mTOR pathway via PI3K [53, 54]. We can reasonably speculate that these events are involved in our experiments as well. Very few of these studies investigated the kinetics of mTOR activation. However, mTOR target’s phosphorylation previously reported by Kenney and colleagues is reminiscent of our findings [52]. Our results show that in hippocampal neurons, mTOR is activated by a transient depolarization and that AIMTOR BRET signal lasts for at least 20 min. However, since AIMTOR signal integrates mTOR and phosphatases activity, this long-lasting substrate phosphorylation could either report mTOR long-lasting activation or phosphatase inhibition during the late phase of neuronal plasticity. Further experiments will be required to decipher the signaling implicated in long-lasting phosphorylation of mTOR targets. Specifically, mTOR activity during plasticity is still an open, very interesting question. An mTOR long-lasting activation may be required in the late phase of neuronal plasticity. Conversely, mTOR activation could also be only transient to initiate mRNA translation in proteins later involved in plasticity. Importantly, this fine regulation of mTOR signaling is lost in the two ASD mouse models tested in the present study. These results are in accordance with a growing body of evidences suggesting the involvement of mTOR dysfunction in neurodevelopmental disorders associated with cognitive impairments. On the one hand, monogenic mutations in genes encoding for the mTORC1 complex or its direct functional regulators lead to the emergence of ASD. “mTORopathies” indeed include monogenic mutations in mTOR itself, TSC1/TSC2 complex, PTEN, or AKT and are found to be associated with ASD [55–57]. On the other hand, synaptopathies associated with ASD, engendered by monogenic mutations of genes coding for proteins involved in the neuronal architecture and/or regulation of the neuronal communication, have reversely been associated with increased mTOR signaling. For example, *Fmr1*^*−/y*^ mutant mice display a high mTOR signaling, an increased protein synthesis, and an excessive and dysregulated mGluR-dependent long-term depression [58–60]. These phenotypes could be reversed by downregulation of mTOR activity [60]. Our own data are corroborating and better refining an excessive and uncontrolled mTORC1 activity in *Fmr1*^*−/y*^ mutant mice. Besides, AIMTOR could help phenotyping mTOR signaling in neurological disorders, as we did for Shank3Δ11 mice. Given that disruption of mTOR and MAPK pathways correlates with severity in idiopathic autism [61], AIMTOR could even serve diagnosis purpose.

## Conclusions

In conclusion, we have developed a potent and specific BRET mTOR biosensor to assess subcellular activities of mTORC1 in living cells. This could help to investigate mTOR dynamics in several cellular contexts and to better phenotype cells harboring various dysfunctions of mTOR signaling.

## Supplementary information

**Additional file 1 : Figure S1**: **A)** Schematic representation of mTORC1 signaling. The multiprotein complex mTORC1 is activated by growth factors and nutriments, notably amino acids. In contrast, mTORC1 is inhibited by energetic stress. Once activated mTORC1 phosphorylates several substrates to induce or inhibit several biological responses. Four specific substrates of mTORC1 are shown: 4EBP1 and S6K1 proteins controlling protein translation; Ulk1 controlling autophagy and TFEB regulating lysosome biogenesis. **B)** Sequences of mTOR target peptides. Shown are the name of the natural mTORC1 substrates and the respective sequence of the target peptide(s) phosphorylated by mTORC1 and tested as biosensors. The amino acid positions phosphorylated by mTORC1 are in bold underlined. **C)** Screening for the best responding biosensor. The histogram represents the BRET intensity – BRET intensity of the “Fasting” condition recorded in live H1299 cells expressing biosensors made with different mTOR target peptides ULK1-T757 (T757) white colored bars, ULK1-S757 (S757) light gray colored bars, 4EBP1 full length (4EBP1) gray colored bars, 4EBP1-S65T70 (S65T70) dark gray colored bars, 4EBP1-T37T46 (T37T46) black colored bars. Cells were incubated in “Fasting” medium (devoid of glutamine and serum) with glutamine (Fasting + GLN) or Rapamycin (Fasting + Rapa) and “rich” medium (supplemented with Glutamine and 10% serum). Each bar of the histogram corresponds to one representative experiment.

**Additional file 2 : Figure S2**: A) S757 biosensor response to mTOR inhibitor: BRET intensity of H1299 cells expressing the S757 biosensor, incubated for 90 min with various concentration of the Torin mTOR inhibitor or with the DMSO vehicle as control. Each dot represents the BRET intensity of a single experiment and the horizontal bar the mean BRET intensity of 3 independent experiments. * *p* < 0.05 and *** *p* < 0.001 compared to the respective DMSO (control) condition and # p < 0.05 compared to the “25 nM” condition. B) In proliferating C2C12 myoblasts expressing AIMTOR, there is no correlation between the BRET intensity and AIMTOR expression level (linear regression fit, R^2^ = 0.02207): each dot represents the mean BRET intensity of a single cell recorded by BRET imaging as a function of AIMTOR expression level quantified by measuring the fluorescence intensity of Ypet. C) Western blots of total lysates of H1299 cells expressing AIMTOR and transfected with siRNA targeting REDD1 (Redd1 siRNA) and control (CTL siRNA) reveal REDD1 protein (above), phosphorylated S6K1 protein (middle), and total protein load (bottom). Fold changes of the intensity of the REDD1 and the phosphorylated S6K1 protein bands relative to the CTL siRNA condition are shown under each blot.

**Additional file 3 : Figure S3**: Additional experiments in C2C12 cells. **A)** Mean BRET intensity of 4 days differentiated C2C12 myotubes expressing cytosolic AIMTOR and treated overnight with DMSO (control, white colored circles) or rapamycin (25 nM and 250 nM, black colored circles). **B)** BRET Imaging of 3 days differentiated C2C12 myotubes expressing cytosolic AIMTOR or cytosolic AIMTOR T757A mutant following rapamycin 250 nM or DMSO (control) overnight incubation. Panel **C-E**: BRET imaging (above) and BRET intensity quantification (below) of 5 days differentiated C2C12 myotubes expressing lysosomal (**C**), nuclear (**D**), or mitochondrial (**E**) AIMTOR treated overnight with Rapamycin (black colored circles) or DMSO (control, white colored circles). Each dot represents the quantification of the BRET intensity recorded in a single cell and the horizontal bars show the mean of BRET intensities obtained from 20 to 25 cells per condition. Mann Whitney statistical analysis with **** *p* < 0.0001 compared to control condition. Scale bar represents 20 μm. Panel **F-I:** Mean BRET intensity of 7 days differentiated C2C12 cells expressing cytosolic AIMTOR (**F**), lysosomal AIMTOR (**G**), nuclear AIMTOR (**H**), and mitochondrial (**I**) AIMTOR, incubated 1 h in medium supplemented with Rapamycin 250 nM (black colored circles), or DMSO (control, white colored circles). Each dots represents the BRET intensity of a single experiment and the horizontal bars show the mean of BRET intensities of 3 independent experiments. For statistical analysis, Wilcoxon two-tailed matched paired sign rank test, ** *p* < 0.01 compared to the control condition.

**Additional file 4.** Individual data values for all experiments in an Excel file. Data sets are sorted by figure.

## Data Availability

Data generated and shown in figures of this study are included in Additional file 4. The datasets generated or analyzed during the current study are available from the corresponding author on reasonable request. AIMTOR and non-responding mutant encoding plasmids are available at Addgene as indicated in the “Material and methods” section.
