# Supplementary material for: AIMTOR, a BRET biosensor for live imaging, reveals subcellular mTOR signaling and dysfunctions
Source: BMC Biol. 2020 Jul 3;18:81. doi: 10.1186/s12915-020-00790-8 (PMC7334845; doi:10.1186/s12915-020-00790-8)
Supplement: Supplementary file 1 — Additional file 1 : Figure S1: A) Schematic representation of mTORC1 signaling. The multiprotein complex mTORC1 is activated by growth factors and nutriments, notably amino acids. In contrast, mTORC1 is inhibited by energetic stress. Once activated mTORC1 phosphorylates several substrates to induce or inhibit several biological responses. Four specific substrates of mTORC1 are shown: 4EBP1 and S6K1 proteins controlling protein translation; Ulk1 controlling autophagy and TFEB regulating lysosome biogenesis. B) Sequences of mTOR target peptides. Shown are the name of the natural mTORC1 substrates and the respective sequence of the target peptide(s) phosphorylated by mTORC1 and tested as biosensors. The amino acid positions phosphorylated by mTORC1 are in bold underlined. C) Screening for the best responding biosensor. The histogram represents the BRET intensity – BRET intensity of the “Fasting” condition recorded in live H1299 cells expressing biosensors made with different mTOR target peptides ULK1-T757 (T757) white colored bars, ULK1-S757 (S757) light gray colored bars, 4EBP1 full length (4EBP1) gray colored bars, 4EBP1-S65T70 (S65T70) dark gray colored bars, 4EBP1-T37T46 (T37T46) black colored bars. Cells were incubated in “Fasting” medium (devoid of glutamine and serum) with glutamine (Fasting + GLN) or Rapamycin (Fasting + Rapa) and “rich” medium (supplemented with Glutamine and 10% serum). Each bar of the histogram corresponds to one representative experiment. [file 12915_2020_790_MOESM1_ESM.pptx]

## Slide 1
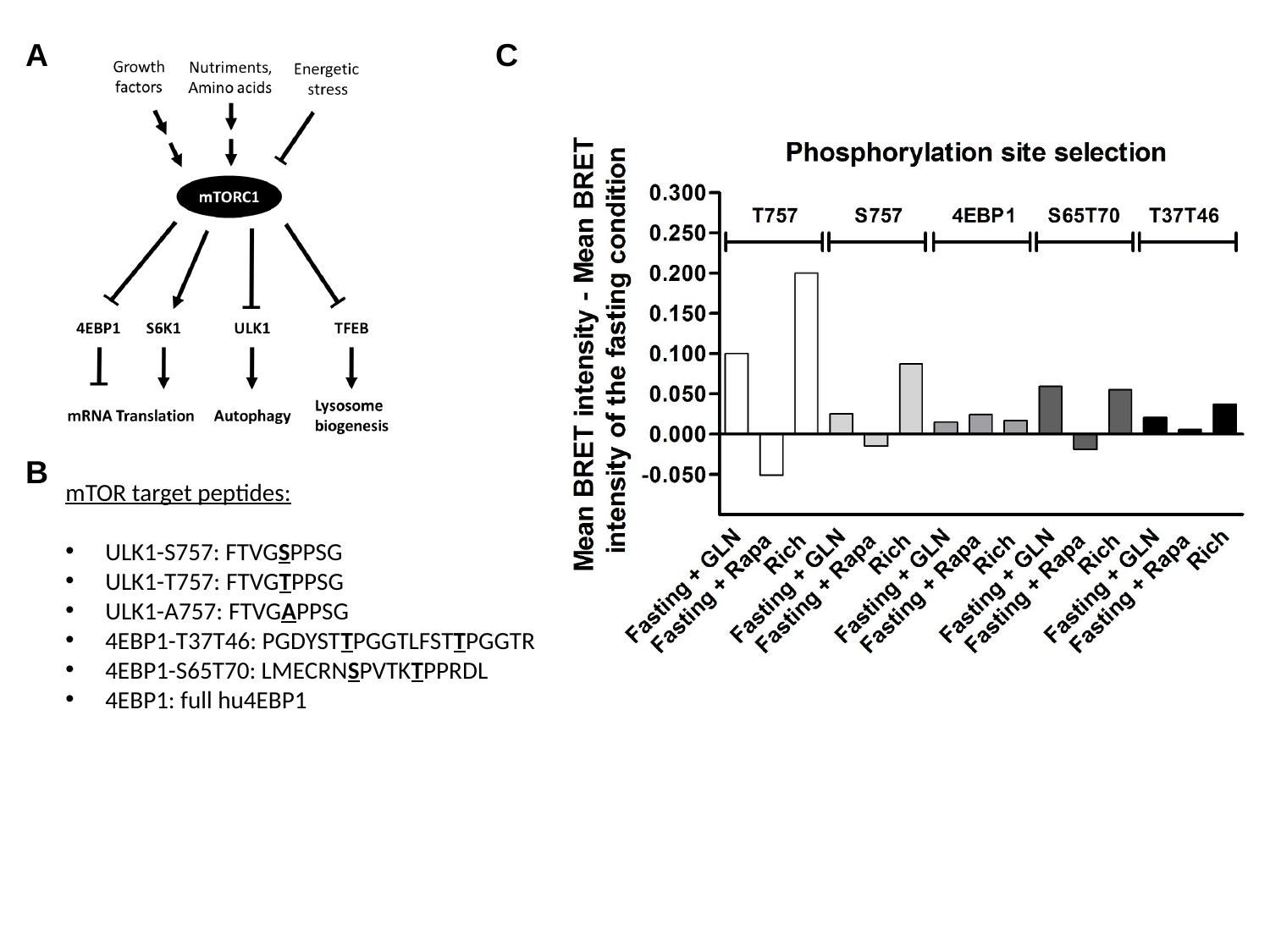

A
C
B
mTOR target peptides:
ULK1-S757: FTVGSPPSG
ULK1-T757: FTVGTPPSG
ULK1-A757: FTVGAPPSG
4EBP1-T37T46: PGDYSTTPGGTLFSTTPGGTR
4EBP1-S65T70: LMECRNSPVTKTPPRDL
4EBP1: full hu4EBP1
