# Supplementary figures and images for: AIMTOR, a BRET biosensor for live imaging, reveals subcellular mTOR signaling and dysfunctions
Source: BMC Biol. 2020 Jul 3;18:81. doi: 10.1186/s12915-020-00790-8 (PMC7334845; doi:10.1186/s12915-020-00790-8)

## Slide 1
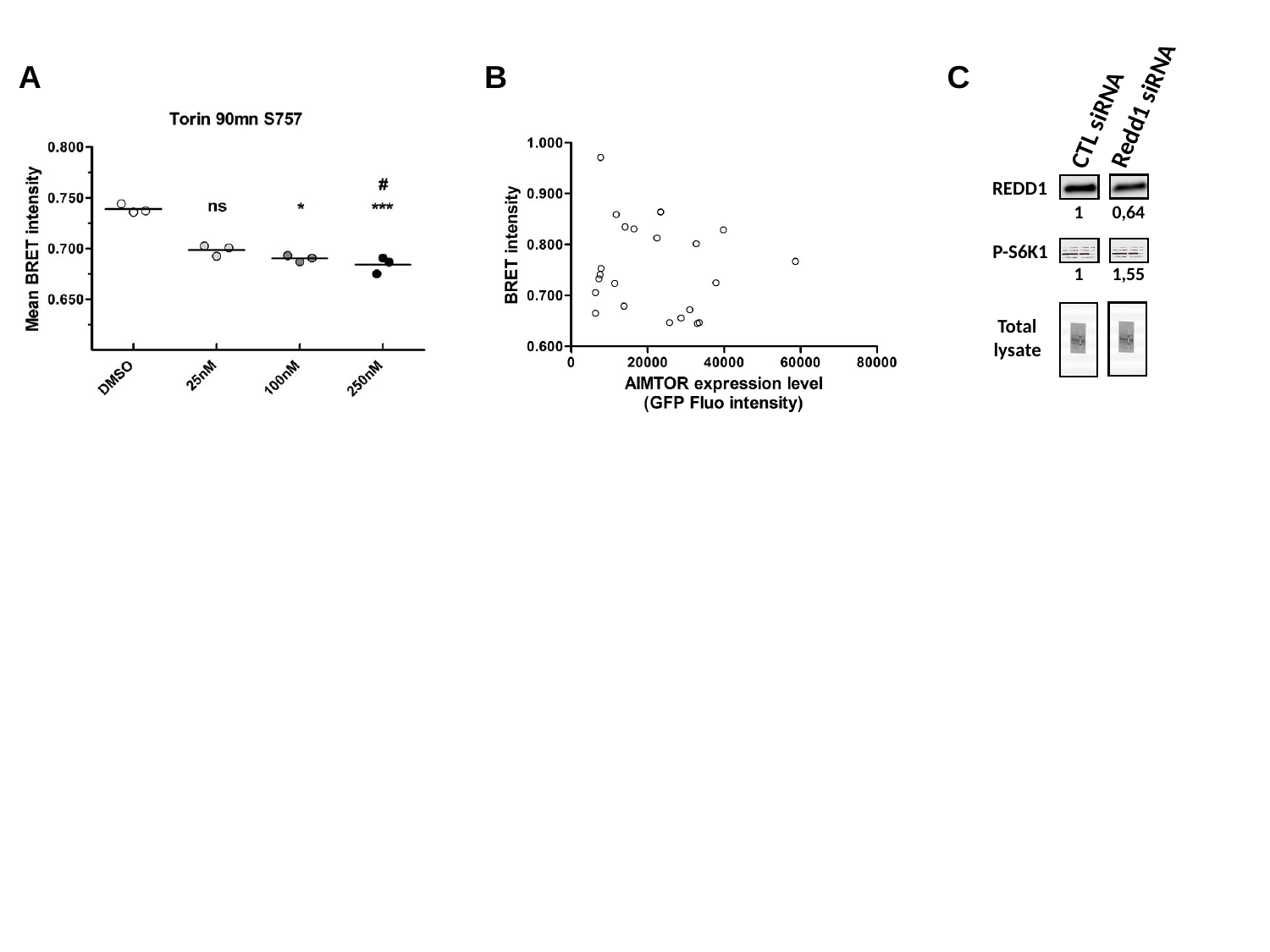

Redd1 siRNA
CTL siRNA
REDD1
1
0,64
P-S6K1
1
1,55
Total
lysate
A
B
 C

Supplement: Supplementary file 2 — Additional file 2 : Figure S2: A) S757 biosensor response to mTOR inhibitor: BRET intensity of H1299 cells expressing the S757 biosensor, incubated for 90 min with various concentration of the Torin mTOR inhibitor or with the DMSO vehicle as control. Each dot represents the BRET intensity of a single experiment and the horizontal bar the mean BRET intensity of 3 independent experiments. * p < 0.05 and *** p < 0.001 compared to the respective DMSO (control) condition and # p < 0.05 compared to the “25 nM” condition. B) In proliferating C2C12 myoblasts expressing AIMTOR, there is no correlation between the BRET intensity and AIMTOR expression level (linear regression fit, R2 = 0.02207): each dot represents the mean BRET intensity of a single cell recorded by BRET imaging as a function of AIMTOR expression level quantified by measuring the fluorescence intensity of Ypet. C) Western blots of total lysates of H1299 cells expressing AIMTOR and transfected with siRNA targeting REDD1 (Redd1 siRNA) and control (CTL siRNA) reveal REDD1 protein (above), phosphorylated S6K1 protein (middle), and total protein load (bottom). Fold changes of the intensity of the REDD1 and the phosphorylated S6K1 protein bands relative to the CTL siRNA condition are shown under each blot. [file 12915_2020_790_MOESM2_ESM.pptx]

## Slide 1
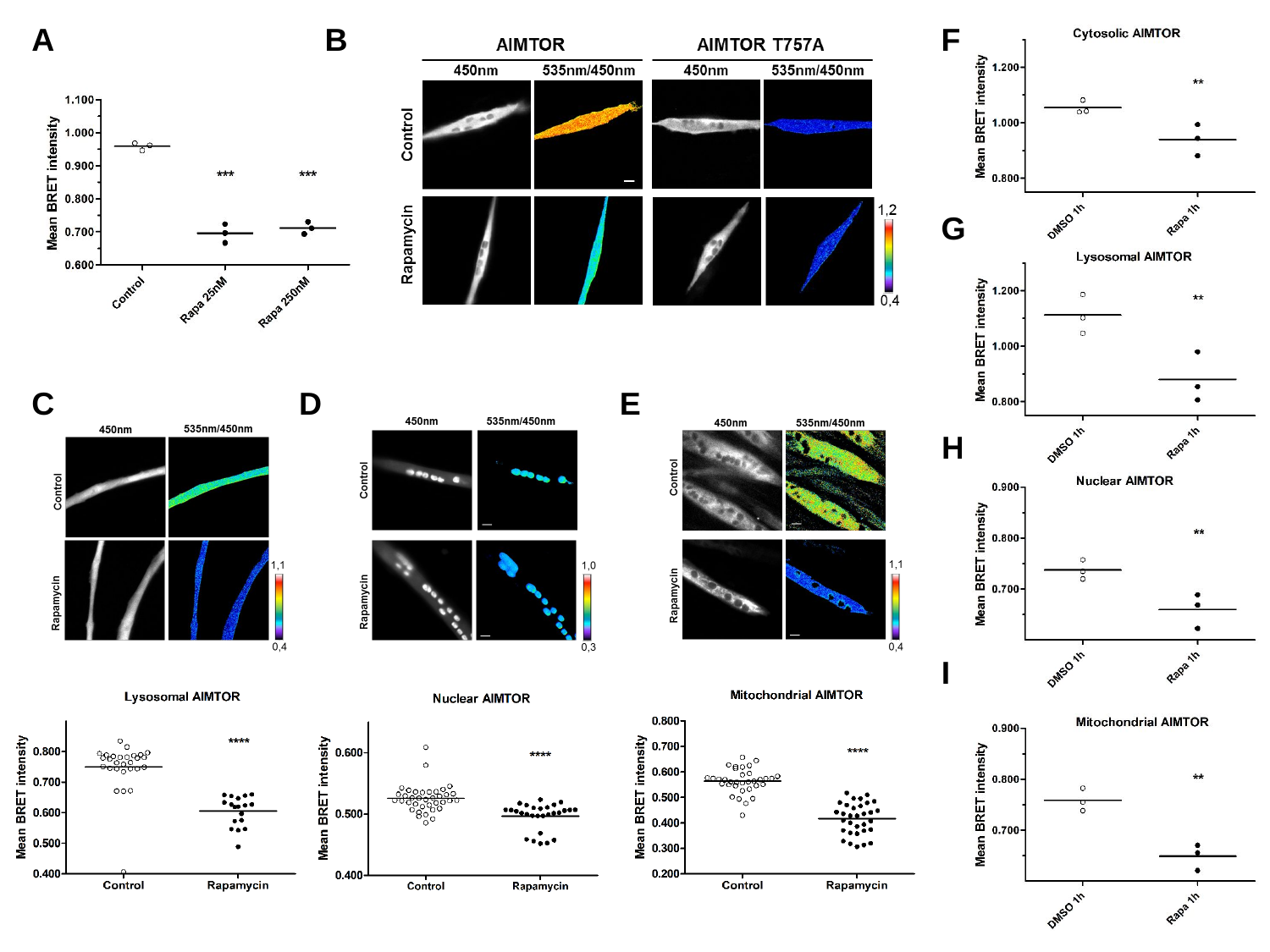

A
B
F
G
C
D
E
H
I

Supplement: Supplementary file 3 — Additional file 3 : Figure S3: Additional experiments in C2C12 cells. A) Mean BRET intensity of 4 days differentiated C2C12 myotubes expressing cytosolic AIMTOR and treated overnight with DMSO (control, white colored circles) or rapamycin (25 nM and 250 nM, black colored circles). B) BRET Imaging of 3 days differentiated C2C12 myotubes expressing cytosolic AIMTOR or cytosolic AIMTOR T757A mutant following rapamycin 250 nM or DMSO (control) overnight incubation. Panel C-E: BRET imaging (above) and BRET intensity quantification (below) of 5 days differentiated C2C12 myotubes expressing lysosomal (C), nuclear (D), or mitochondrial (E) AIMTOR treated overnight with Rapamycin (black colored circles) or DMSO (control, white colored circles). Each dot represents the quantification of the BRET intensity recorded in a single cell and the horizontal bars show the mean of BRET intensities obtained from 20 to 25 cells per condition. Mann Whitney statistical analysis with **** p < 0.0001 compared to control condition. Scale bar represents 20 μm. Panel F-I: Mean BRET intensity of 7 days differentiated C2C12 cells expressing cytosolic AIMTOR (F), lysosomal AIMTOR (G), nuclear AIMTOR (H), and mitochondrial (I) AIMTOR, incubated 1 h in medium supplemented with Rapamycin 250 nM (black colored circles), or DMSO (control, white colored circles). Each dots represents the BRET intensity of a single experiment and the horizontal bars show the mean of BRET intensities of 3 independent experiments. For statistical analysis, Wilcoxon two-tailed matched paired sign rank test, ** p < 0.01 compared to the control condition. [file 12915_2020_790_MOESM3_ESM.pptx]
